# Brightly Visualizing Pancreatic Cancer Margins in Orthotopic Mouse Models with an Anti-CA19-9 Antibody Conjugated to a Near-Infrared Fluorophore

**DOI:** 10.3390/cancers17162617

**Published:** 2025-08-10

**Authors:** Kristin E. Cox, Javier Bravo, Sunidhi Jaiswal, Siamak Amirfakhri, Thinzar M. Lwin, Abhijit Aithal, Sumbal Talib, Lily J. Jih, Aylin Din Parast Saleh, Keita Kobayashi, Kavita Mallya, Maneesh Jain, Robert M. Hoffman, Aaron M. Mohs, Surinder K. Batra, Michael Bouvet

**Affiliations:** 1 Department of Surgery, University of California San Diego, La Jolla, CA 92092, USAjbquintana@health.ucsd.edu (J.B.); s3jaiswal@health.ucsd.edu (S.J.); siamirfakhri@health.ucsd.edu (S.A.); kjkobayashi@ucsd.edu (K.K.); rhoffman@health.ucsd.edu (R.M.H.); 2 VA San Diego Healthcare System, La Jolla, CA 92161, USA; lily.jih@va.gov; 3 Department of Surgical Oncology, City of Hope National Medical Center, Duarte, CA 91010, USA; tlwin@coh.org; 4 Department of Biochemistry and Molecular Biology, University of Nebraska Medical Center, Omaha, NE 68198, USA; abhijit.aithal@unmc.edu (A.A.); kmallya@unmc.edu (K.M.); mjain@unmc.edu (M.J.); sbatra@unmc.edu (S.K.B.); 5 Fred and Pamela Buffett Cancer Center, University of Nebraska Medical Center, Omaha, NE 68198, USA; stalib@unmc.edu (S.T.); aaron.mohs@unmc.edu (A.M.M.); 6 Department of Pharmaceutical Sciences, University of Nebraska Medical Center, Omaha, NE 68198, USA; 7 AntiCancer Inc., San Diego, CA 92111, USA

**Keywords:** CA19-9, fluorescence-guided surgery, fluorescent imaging, orthotopic mouse model, pancreatic cancer, SPY, tumor marker

## Abstract

**Background/Purpose:** The only potentially curative procedure for pancreatic cancer is R0 resection, which is difficult to achieve due to poorly defined tumor margins. In the present study, we used an anti-CA19-9 antibody conjugated to a near-infrared fluorophore in orthotopic mouse models to target and visualize pancreatic cancer. **Methods:** Orthotopic models of the human pancreatic cancer cell lines SW1990 and BxPC3 were established by implanting tumor fragments into the pancreas of athymic nude mice. Anti-CA19-9 and control IgG were conjugated with IRDye800CW. Mice received 50 µg of CA19-9–IRDye800CW or IgG-IRDye800CW via tail-vein injection and were imaged after 72 h. MIA PaCa-2, a CA19-9-negative cell line, was used in subcutaneous models to assess targeting specificity. **Results:** Using the LI-COR Pearl imaging system in the SW1990 model, the tumor-to-pancreas ratio (TPR) was 4.51 (±0.74), and the tumor to the liver ratio (TLR) was 3.05 (±0.60) with CA19-9-IRDye800CW, while the TPR was 1.67 (±0.16) and the TLR was 0.95 (±0.05) for the non-specific control IgG–IRDye800CW. Using a clinically available fluorescence laparoscope, CA19-9-1RDye800CW demonstrated a TPR of 2.34 (±0.44) and a TLR of 2.23 (±0.49), compared to 1.11 (±0.13) and 0.69 (±0.07), respectively, for IgG-IRDye800CW in the SW1990 orthotopic model. In the BxPC3 models, the TPR was 3.82 (±0.55) and the TLR was 4.13 (±0.77) for CA19-9-IRDye800CW compared to 2.40 (±0.31) and 1.49 (±0.23), respectively, for IgG-IRDye800CW. **Conclusions:** CA19-9-IRDye800CW provided specific in vivo targeting of two human pancreatic cancer cell lines in orthotopic nude mouse models with superior TPRs and TLRs compared to IgG-IRDye800CW. This tumor-specific fluorescent CA19-9 antibody is a promising clinical tool for improved visualization of pancreatic cancer.

## Simple Summary

Pancreatic cancer is one of the deadliest cancers and surgery offers the only real chance for a cure. However, it is often difficult for surgeons to clearly see the margins and perform a complete resection. In the present study, we used an anti-CA19-9 antibody conjugated to a near-infrared fluorophore to label pancreatic cancer in orthotopic nude mouse models. The fluorescence signal was strong, specific, and visible using a clinical fluorescence laparoscope, suggesting it could help surgeons better detect and remove tumors. These results support the potential use of this technique to improve outcomes in patients with pancreatic cancer.

## 1. Introduction

Pancreatic ductal adenocarcinoma (PDAC) remains one of the most lethal malignancies and is currently the third leading cause of cancer-related deaths, with a 5-year survival rate of 13% [1,2]. The disease is typically diagnosed at a late stage due to non-specific or absent early symptoms, and approximately 80% of patients present with locally advanced or metastatic disease at time of diagnosis [3]. Its aggressive biology, rapid progression, and resistance to systemic therapies limit effective treatment options. With PDAC prevalence rates estimated to increase by 2040 [4], optimizing surgical approaches is critical, as R0 resection remains the only potentially curative option [5,6]. However, surgeons often face significant difficulty in visualizing tumor margins and achieving complete resection in pancreatic cancer due to several factors. The pancreas lies in a complex anatomical location—retroperitoneal and closely associated with critical vascular structures such as the celiac axis, superior mesenteric artery, and portal vein—making surgical access and dissection technically demanding [7,8]. Furthermore, pancreatic tumors are highly aggressive and infiltrative, frequently inducing a dense desmoplastic stromal reaction. This fibrotic response, combined with minimal visual distinction between tumor and normal tissue under standard white light, complicates intraoperative margin assessment [9]. These challenges contribute to a high rate of incomplete resections and highlight the potential value of fluorescence-guided surgery (FGS) to enhance tumor visualization and surgical precision [10].

FGS has emerged as a technique that allows surgeons to precisely identify tumors, distinguish tumors from surrounding tissues, and recognize peritoneal metastases [11,12]. FGS can potentially enhance surgical resection, improving outcomes and overall patient survival. FGS requires tumor-targeting molecules, typically antibodies, to be conjugated with fluorescent dyes and injected intravenously. A near-infrared (NIR) fluorescence imaging system capable of exciting these fluorophores captures their emission, which is projected back to the surgeon in real-time.

Carbohydrate antigen 19-9 (CA19-9) is a common tumor marker expressed in approximately 80% of patients with PDAC and is localized to both the cytoplasm and membrane of tumors cells [13,14,15]. Although it is primarily used as a prognostic biomarker, and its serum levels correlate with disease burden [16], CA19-9 has known limitations. It can yield false-positive results in benign conditions such as cholangitis and pancreatitis, and a subset of PDAC patients do not express CA19-9 [17]. Despite these limitations, in patients who do express CA19-9, it represents a promising molecular target for tumor-specific imaging with fluorescently labeled antibodies [18].

Although previous studies have utilized fluorescent antibodies targeting tumor markers such as CEA and EGFR, many have used visible-spectrum dyes like Alexa Fluor 488 or DyLight 650 [19,20,21], which are suboptimal for clinical translation due to limited tissue penetration and lack of compatibility with surgical imaging platforms. More recently, IRDye800CW linked to tumor specific antibodies has been used in both preclinical and early clinical studies due to its NIR emission and safety profile [22,23,24]. In the present study, we extend this work by conjugating IRDye800CW to a CA19-9-specific monoclonal antibody (NS19.9) and evaluating its potential in subcutaneous and orthotopic PDAC models using both preclinical (Pearl) and clinical (SPY) imaging systems. Several antibody–fluorophore conjugates have already entered clinical trials for PDAC, including agents targeting CEA, EGFR, and VEGF (Table 1). These ongoing efforts highlight growing interest in molecularly targeted intraoperative imaging and provide a clinical framework into which fluorescent CA19-9 antibody–based strategies may be incorporated.

To our knowledge, this is the first study to demonstrate the tumor-labeling capability of anti-CA19-9-IRDye800CW using an FDA-cleared imaging system used in clinical practice, emphasizing its translational relevance. In this study, we evaluate the potential of fluorescent anti-CA19-9 antibodies for selective visualization of orthotopic xenograft tumors using NIR and SPY fluorescence. By demonstrating tumor-specific labeling with a clinically compatible imaging system, the present study bridges a key gap between preclinical imaging and clinical application and supports the feasibility of applying FGS to improve resection outcomes in patients with PDAC.

## 2. Materials and Methods

*Mice.* Athymic nude mice 4–6 weeks of age were purchased from the Jackson Laboratory (Bar Harbor, ME, USA). Mice were selected without applying specific inclusion or exclusion criteria. Randomization was not used to allocate mice to control and treatment groups. The mice were kept in a barrier facility and fed an autoclaved laboratory-approved diet. For anesthesia prior to surgical procedures, an intraperitoneal (IP) injection of a xylazine, ketamine, and phosphate-buffered saline (PBS) mixture was administered. For the final analysis, mice were euthanized with isoflurane inhalation and cervical dislocation. The experiment was approved by the San Diego Veterans Administration Medical Center Institutional Animal Care and Use Committee. This study follows to the ARRIVE 2.0 guidelines to ensure comprehensive and transparent reporting of our animal research.

*Antibody conjugation and characterization.* A monoclonal antibody, specific for CA19-9 (NS19.9), was purified from hybridoma culture and conjugated to a near-infrared dye IRDye800CW (LI-COR Biosciences, Lincoln, NE, USA) according to instructions provided by the manufacturer. The mix was then incubated for 2 h, yielding CA19-9-IRDye800CW. Excess unbound dye was removed using ZEBA spin desalting columns (ThermoFisher Scientific, Waltham, MA, USA) via size-exclusion chromatography. This method separates molecules based on size, allowing larger antibody–dye conjugates to pass through while retaining smaller unbound dye molecules within the column matrix during centrifugation. The filtered conjugate was stored at 4 degrees Celsius. Mouse IgG antibody was also conjugated to IRDye800 CW as a control, yielding IgG-IR800. The dye-to-protein (D/P) ratio and protein concentration were quantified using spectra obtained with an Evolution 220 absorbance spectrophotometer (ThermoFisher Scientific, Waltham, MA). The fluorescence spectrum of the conjugates was obtained using a FluoroMax 4 spectrofluorometer (Horiba Scientific; Irvine, CA, USA). The conjugates were administered to mice via tail vein injection.

*Cell lines.* Three different human pancreatic cancer cell lines were used in the present study: SW1990, BxPC3, and MIA PaCa-2. All cell lines were obtained from the American Type Culture Collection (ATCC) (Manassas, VA, USA).

*Establishment of orthotopic nude mouse models of pancreatic cancer.* Orthotopic models of both SW1990 and BxPC3 cell lines were established using surgical orthotopic implantation (SOI) through the following steps: (1) IP anesthesia injection, (2) disinfection of mice ventral area with 70% ethanol solution, (3) entry into the abdominal cavity via left flank incision, (4) implantation of tumor fragments (2 mm^3^ SW1990 or BxPC3) into the middle portion of the pancreas, and (5) closure of the incision with 6–0 Vicryl interrupted sutures (Ethicon Inc., Somerville, NJ, USA) [29]. Postoperative pain was managed with the subcutaneous administration of 100 µL buprenorphine solution (dosage: 0.05 mg/kg). For the SW1990 group, a total of 10 mice were studied; for the BxPC3 group, a total of 12 mice were studied. At least 10 mice were used in each group to increase the statistical power of the experiment.

*Establishment of subcutaneous mouse models of pancreatic cancer.* Subcutaneous models were created with an internal control group. Tumor fragments of ~2 mm^3^ of CA19-9-positive SW1990 and CA19-9-negative MIA PaCa-2 cell lines were implanted subcutaneously into the posterior flanks of 3 nude mice. Three mice were included in the CA19-9 negative control group, providing sufficient data for comparison while minimizing unnecessary animal use given the predictable and controlled outcome. Tumors were allowed to grow until they reached 1 cm in size, which occurred after approximately 3 weeks. MIA PaCa-2 was used exclusively in subcutaneous models as a CA19-9-negative control. To avoid unnecessary animal use, orthotopic implantation was not performed with this cell line, as CA19-9-IR800 was not expected to accumulate in these tumors due to the absence of antigen expression.

*Fluorescent antibody administration.* In the SW1990 orthotopic model group (*n* = 10), 6 mice received 50 µg CA19-9-IR800 and 4 mice received 50 µg IgG-IRDye800CW via tail vein injection. In the BxPC3 group (*n* = 12), 8 mice received 50 µg CA19-9-IRDye800CW and 4 mice received 50 µg IgG-IRDye800CW. In the MIA PaCa-2/SW1990 subcutaneous group (*n* = 3), 3 mice received only 50 µg anti-CA19-9-IRDye800CW. After 72 h, the mice were anesthetized with isoflurane and euthanized by cervical dislocation. A midline laparotomy was performed on the orthotopic models to allow bright light, near-infrared (NIR) imaging, and SPY fluorescence imaging of the tumors and other intra-abdominal organs. A total of 25 mice received antibody injections for imaging. All mice involved were included in the final analysis. To minimize potential confounders, mice from each treatment and control group were housed in separate, clearly labeled cages according to the type of tumor implanted. This approach helped ensure consistent group identification and reduced the risk of cross-contamination or misclassification.

*Imaging.* The Pearl Trilogy Imaging System (LI-COR Biosciences, Lincoln, NE, USA), capable of 785 nm excitation and 820 nm emission (800 channel), was used for NIR imaging. The same brightness and contrast settings were used for all imaging experiments. Regions of interest were drawn, using the system’s software, around the tumors, normal pancreas, and liver while viewing bright light images. The mean fluorescence intensity (mFI) of the NIR signals was then calculated for each tumor. To determine if the molecule was able to specifically label the tumor, we calculated tumor-to-pancreas ratios (TPRs) and tumor-to-liver ratios (TLRs) by dividing the mFI of the tumor by the mFI of the nearby normal pancreas for TPRs and by the mFI of the liver for TLRs. The Stryker 1688 AIM 4 K Platform (Stryker Corporation, Kalamazoo, MI, USA) was used for SPY fluorescence imaging of the SW1990 orthotopic mouse models. These images were then analyzed using the ImageJ software (https://imagej.net/ij/, NIH, Bethesda, MD, USA) to obtain pixel values to calculate TPRs and TLRs. For subcutaneous tumors, mice were imaged at 24, 48, 72, and 96 h. The MIA PaCa-2/SW1990 internal control group also underwent fluorescence imaging. A tumor-to-background ratio (TBR) was calculated using the mFI of the skin as the background. The mice in the subcutaneous model were imaged at various time points to determine which time point resulted in the highest mFI. The orthotopic model was then imaged at this time point for optimal imaging results. Orthotopic mouse models cannot be imaged at various time points, as the mouse is sacrificed prior to orthotopic imaging, whereas the mice in subcutaneous models can be lightly anesthetized for imaging at various time points. The 72 h time point was chosen for orthotopic imaging based on optimization studies and preliminary data showing peak TBRs at this time.

*Statistical Analysis.* Statistical analysis was performed using R software (https://www.r-project.org/, Free Software Foundation, Boston, MA, USA). Each of the groups (SW1990 and BxPC3 models treated with CA19-9-IRDye800CW or IgG-IRDye800CW) was found to be normally distributed using a Shapiro test. A Student’s *t*-test with two tails was performed to compare the TPRs, TLRs, and TBRs of CA19-9-IRDye800CW and IgG-IRDye800CW in the pancreatic cancer unde mouse models. A *p*-value of <0.05 was used as a predetermined cutoff for statistical significance. The investigators were aware of group allocation during all stages of the experiment, including allocation, conduct, outcome assessment, and data analysis.

## 3. Results

### 3.1. Antibody–Dye Conjugate Excitation and Emission Wave Lengths

**Figure 1:**
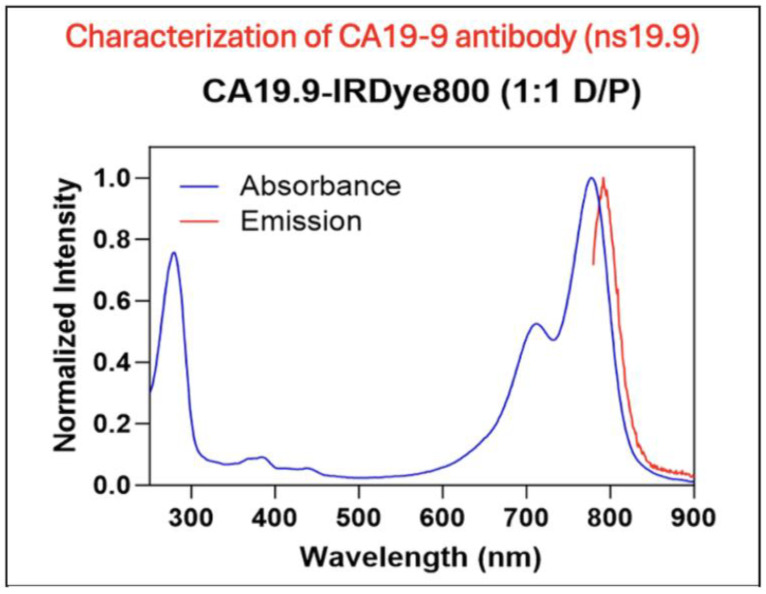
Characterization of the anti-CA19-9 antibody. Excitation and emission spectra of the CA19-9-IR800 conjugate. CA19-9-IR800 had an excitation peak at 775 nm and an emission peak at 792 nm.

The CA19-9–IRDye800CW antibody–dye conjugate was analyzed on a spectrophotometer. The excitation and emission profiles of the molecule are shown in Figure 1. CA19-9-IRDye800CW had an excitation peak at 775 nm and an emission peak at 792 nm. The conjugate demonstrated a dye-to-protein ratio of 1.1.

### 3.2. CA19-9-IRDye800CW Shows Superior Tumor Labeling Versus Control in Orthotopic Nude Mouse Models of Pancreatic Cancer

**Figure 2:**
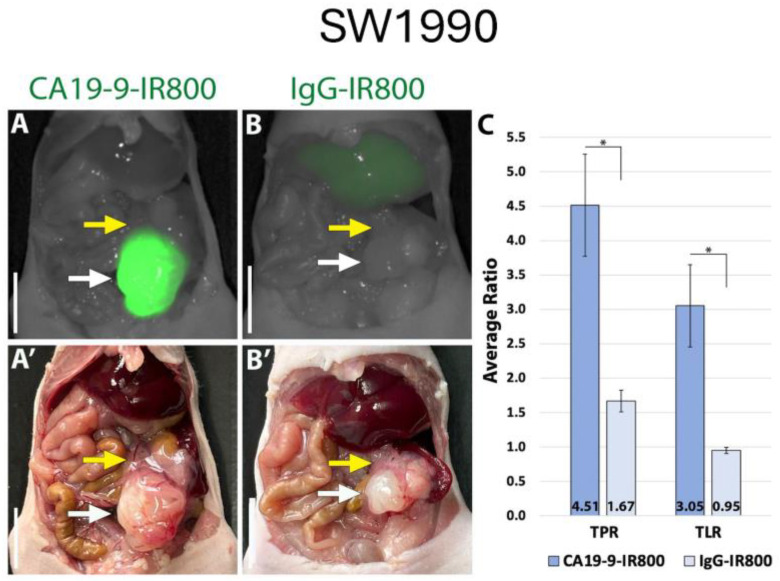
CA19-9-IRDye800CW brightly targets pancreatic cancer cell line SW1990 in orthotopic mouse models. (**A**) NIR and (**A′**) bright light imaging of pancreatic tumors labeled with 50 µg CA19-9-IRDye800CW. (**B**) NIR and (**B′**) bright light imaging of pancreatic tumors demonstrating non-specific labeling with 50 µg IgG-IRDye800CW. (**C**) Average tumor-to-pancreas ratios (TPRs) and tumor-to-liver ratios (TLRs) of mice treated with CA19-9-IRDye800CW or IgG-IRDye800CW. White arrow: tumor, yellow arrow: normal pancreas. Scale bar: 1 cm, * = *p*-value < 0.05.

**Figure 3:**
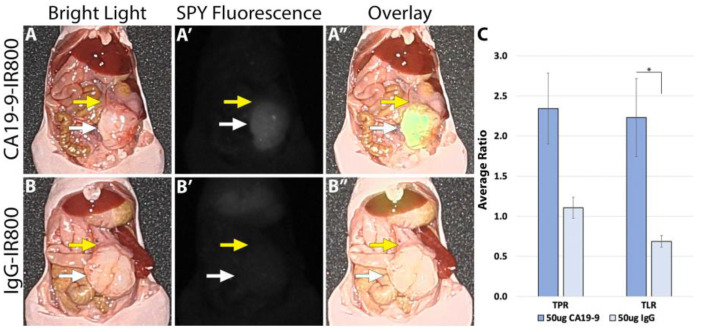
SW1990 cell line orthotopic mouse model of pancreatic cancer labeled with 50 µg CA19-9-IRDye800CW. (**A**) Bright light image, (**A′**) SPY fluorescence, and (**A″**) color overlay modes on the Stryker 1688 laparoscopic imaging device identifying tumors with clear margins after administration of 50 µg CA19-9-IRDye800CW. (**B**) Bright light image, (**B′**) SPY fluorescence, and (**B″**) overlay modes on the Stryker 1688 laparoscopic imaging device with no specific targeting after 50 µg IgG-IRDye800CW administration. White arrow: tumor, yellow arrow: normal pancreas. Scale bar: 1 cm, * = *p*-value < 0.05.

**Figure 4:**
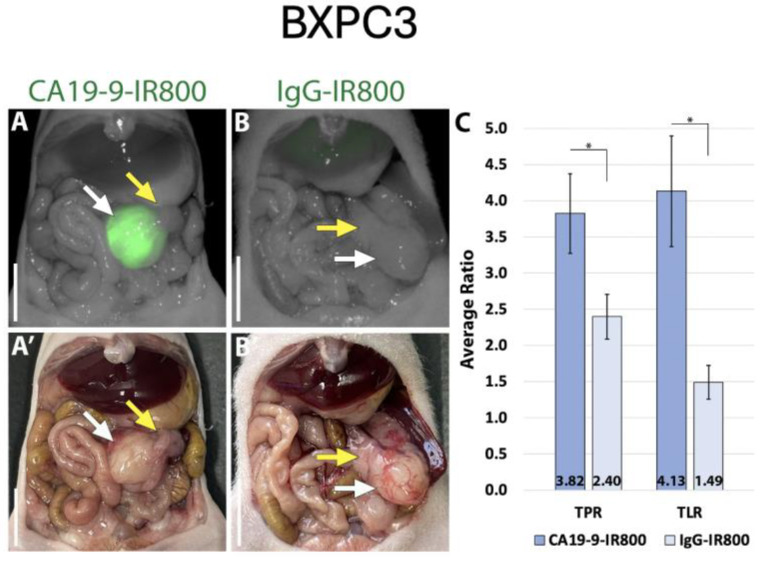
CA19-9-IRDye800CW brightly targets pancreatic cancer cell line BxPC3 in orthotopic mouse models. (**A**) NIR and (**A’**) bright light imaging of pancreatic tumor labeled with 50 µg CA19-9-IRDye800CW. (**B**) NIR and (**B’**) bright light imaging of pancreatic tumor and non-specific labeling with 50 µg IgG-IRDye800CW. (**C**) Average tumor-to-pancreas ratios (TPRs) and tumor-to-liver ratios (TLRs) of those treated with CA19-9-IRDye800CW or IgG-IRDye800CW. White arrow: tumor, yellow arrow: normal pancreas. Scale bar: 1 cm, * = *p*-value < 0.05.

Using the LI-COR Pearl imaging system in the orthotopic models of SW1990 labelled with CA19-9-IRDye800CW, the TPR was 4.51 (±0.74) compared to 1.67 (±0.16) for IgG-IRDye800CW (*p*-value 0.011). The tumor-to-liver ratio (TLR) was 3.05 (±0.60) compared to 0.95 (±0.05) for IgG-IRDye800CW (*p*-value 0.017) (Figure 2). When imaged with SPY fluorescence, the CA19-9-IRDye800CW conjugate demonstrated a TPR of 2.34 (±0.44) and a TLR of 2.23 (±0.49), compared to 1.11 (±0.13) and 0.69 (±0.07) in the IgG-IRDye800CW group (*p*-values 0.053 and 0.031, respectively, as seen in Figure 3). In the BxPC3 models labelled with CA19-9-IRDye800CW, the TPR was 3.82 (±0.55) and the TLR was 4.13 (±0.77) compared to 2.40 (±0.31) and 1.49 (±0.23), respectively, in the IgG-IRDye800CW group (*p*-values 0.047 and 0.014, respectively) (Figure 4).

### 3.3. Biodistribution Studies of CA19-9-IRDye800CW Demonstrate Specific Tumor Labeling

**Figure 5:**
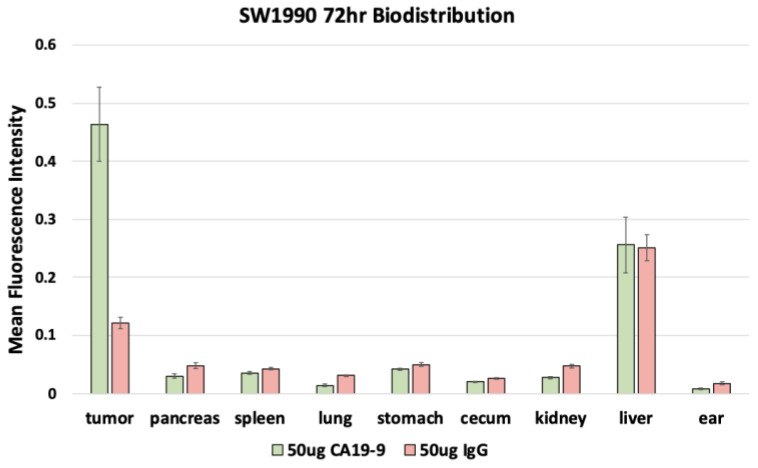
Fluorescence biodistribution of anti-CA19-9-IRDye800CW (*n* = 6) and IgG-IRDye800CW (*n* = 4) in different organs of the orthotopic SW1990 pancreatic cancer cell line mouse models.

**Figure 6:**
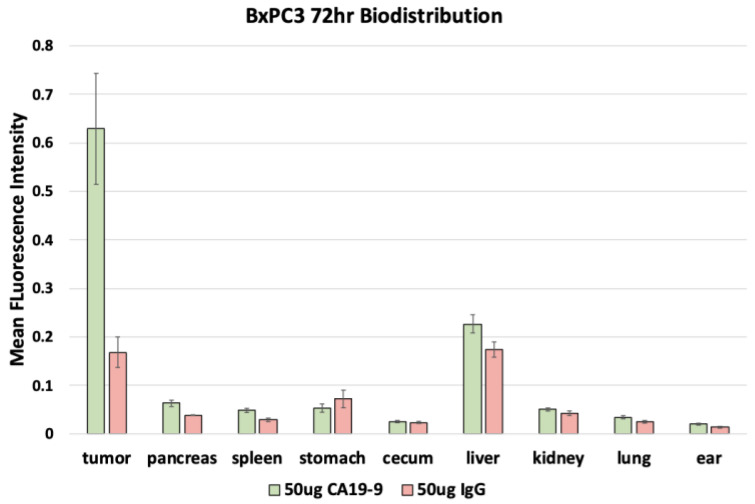
Fluorescence biodistribution of anti-CA19-9-IRDye800CW (*n* = 6) and IgG-IRDye800 (*n* = 4) in different organs of the orthotopic BxPC3 pancreatic cancer cell line mouse models.

After euthanasia, the mice underwent necropsy to perform fluorescence biodistribution analysis. Mice with SW1990 tumors injected with 50 µg CA19-9-IRDye800CW demonstrated the highest mean fluorescence intensity (mFI) in the tumors (0.464 ± 0.064); mice injected with 50 µg IgG-IRDye800CW demonstrated the highest mFI in the liver (0.251 ± 0.022) with relatively low levels seen in the tumors (Figure 5). Similarly, mice with BxPC3 tumors injected with 50 µg CA19-9-IRDye800CW also demonstrated the highest mFI in the tumors (0.629 ± 0.114); mice injected with 50 µg IgG-IRDye800CW demonstrated the highest mFI in the liver (0.173 ± 0.016) with relatively low levels seen in the tumors (Figure 6). In both groups, minimal fluorescence signal was detected in the normal pancreas, spleen, stomach, cecum, kidney, lung, and skin. Background signal in normal tissues was minimal, although some liver uptake was observed with CA19-9-IRDye800CW and IgG-IRDye800CW, consistent with hepatic clearance of antibodies. The normal pancreas showed low levels of background fluorescence across all models, supporting specificity of tumor labeling.

### 3.4. Subcutaneous Imaging Demonstrates CA19-9-IRDye800CW Binds Selectively to CA19-9-Expressing Tumors

**Figure 7:**
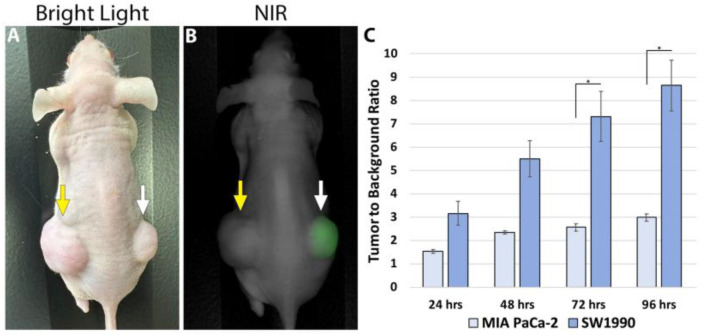
(**A**) Bright light and (**B**) NIR imaging of subcutaneous pancreatic cancer tumors. The CA19-9-negative MIA PaCa-2 tumor in the left flank was not targeted and showed no fluorescence (yellow arrow). CA19-9-IRDye800CW brightly targeted the CA19-9-positive SW1990 tumors in the right flank (white arrow). (**C**) Average TBRs of subcutaneous SW1990 and MIA PaCa-2 tumors over time after tail vein injection of 50 µg CA19-9-IRDye800CW. The highest TBR for the SW1990 tumors was at 96 h (* *p* = 0.04).

Imaging was performed on mice bearing subcutaneous SW1990 and MIA PaCa-2 tumors (which are known to lack CA19-9 expression) at 24, 48, 72, and 96 h after receiving 50 µg CA19-9-IRDye800CW using the LI-COR Pearl Trilogy imaging system (Figure 7). The mean TBR for the SW1990 tumors was significantly higher than for MIA PaCa-2 tumors, with statistical significance reached at the 72 and 96 h time points (*p*-values 0.045 and 0.033, respectively). Both cell lines exhibited peak TBRs at 96 h; the SW1990 TBR at 96 h was 8.63 ± 1.086, while the MiaPaCa2 TBR was 2.98 ± 0.155 (*p* = 0.03). TBRs increased over time in SW1990 subcutaneous tumors but not MIA PaCa-2 subcutaneous tumors (Figure 7).

### 3.5. Immunohistochemical Staining of CA19-9 Expression in Histologic Sections

**Figure 8:**
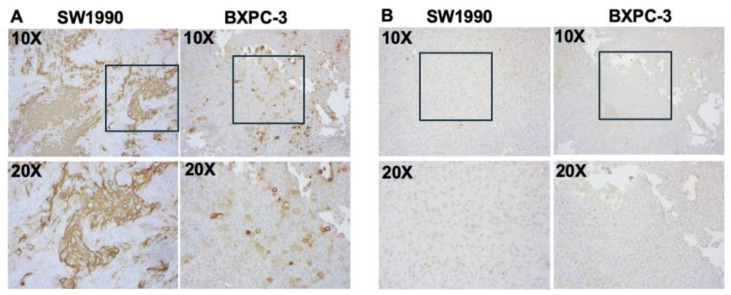
Immunohistochemical staining of CA19-9 expression in xenografts of indicated cell lines. Serial sections were incubated with (**A**) NS19-9 (2 µg/mL) or (**B**) IgG (2 µg/mL) at 4 °C followed by peroxidase labeled secondary antibody and subsequent detection of signal using DAB substrate (Vector Universal Staining KitVector Laboratories, Inc., Newark, CA, USA). Images taken at 10× and 20× are provided. The 20× images correspond to regions outlined by boxes in the respective 10× images.

**Figure 9:**
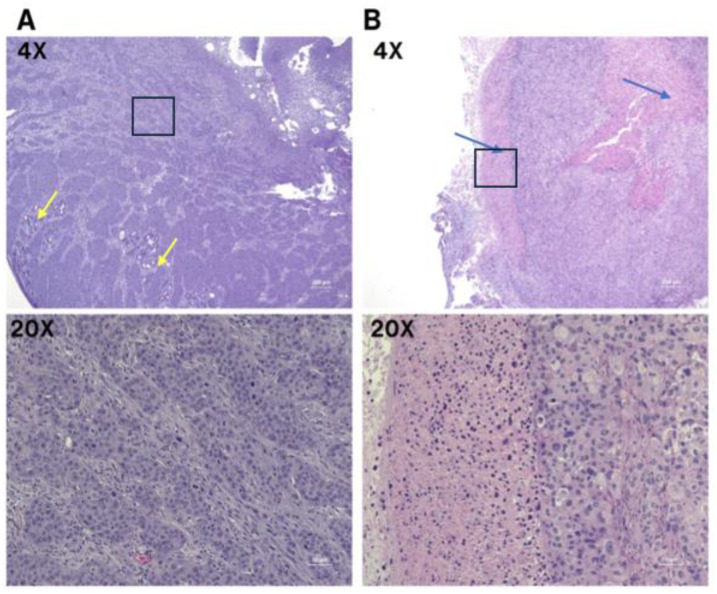
Microscopic images of poorly differentiated pancreatic ductal adenocarcinoma with H&E staining. (**A**) BxPC3 cell line; predominately sheets and nests of infiltrating tumor with focal glandular differentiation, yellow arrows (4×). High power view of poorly differentiated tumor cells with prominent nucleoli and abundant mitosis (20×). (**B**) SW1990 cell line; poorly differentiated pancreatic ductal adenocarcinoma with areas of necrosis, blue arrows (4×). High power view of pleomorphic tumor cells and adjacent necrosis (20×). The 20× images correspond to regions outlined by boxes in the respective 10× images.

CA19-9 staining was observed in both the SW1990 and BxPC3 pancreatic tumors (Figure 8A). Tumors treated with the isotype control IgG-IR800 antibody showed no specific staining (Figure 8B). Hematoxylin and eosin staining was performed for both SW1990 and BxPC3 tumors and confirmed poorly differentiated pancreatic cancer (Figure 9).

## 4. Discussion

The only effective treatment for pancreatic cancer is an R0 resection, which is very difficult to achieve because visualizing tumor margins can be challenging [30,31]. The present study used CA19-9-IRDye800CW in subcutaneous and orthotopic models of pancreatic cancer to brightly visualize pancreatic cancer tumors. Both cell lines with positive CA19-9 expression profiles, SW1990 and BxPC3, were targeted brightly under NIR fluorescence with excellent TBRs.

CA19-9 is the most widely used serum biomarker for pancreatic cancer, primarily aiding in disease monitoring, prognosis, and assessing treatment response [16]. In addition to its secretion into the bloodstream, CA19-9 has been shown to be present within the cytoplasm and on the membrane of pancreatic adenocarcinoma cells [32,33], making it a promising potential antigen for targeted tumor imaging. Houghton et al. targeted CA19-9 in pre-treatment imaging for enhanced tumor visualization [34].

Mouse models of pancreatic cancer can be used to evaluate novel therapeutic and diagnostic agents [35]. McElroy et al. used a monoclonal antibody to CA19-9 conjugated to an AlexaFluor 488 dye to image primary and metastatic pancreatic cancer for surgical navigation [19]. Alexa Fluor 488 is a visible-spectrum fluorophore, with excitation and emission peaks near 495 nm and 525 nm, respectively. In contrast, the present study utilizes a near-infrared (NIR) fluorophore detectable by clinically available laparoscopic systems, making it more suitable for clinical translation. Hiroshima et al. used an anti-CA19-9 antibody conjugated to DyLight 650 dye to demonstrate that metastatic recurrence in pancreatic cancer patient-derived orthotopic xenograft (PDOX) nude mouse model was inhibited by neoadjuvant chemotherapy in combination with fluorescence-guided surgery [21]. In this study, a surgical laparoscope was also assessed, showing encouraging potential for clinical application, in addition to confirming the affinity of CA19-9-IRDye800CW to two different PDAC cell lines using NIR small animal imaging. Metildi et al. established that FGS for pancreatic cancer reduced tumor burden and improved overall survival in mouse models using fluorescently labeled human pancreatic cancer cell lines using an AlexaFluor 488 Dye (ThermoFisher Scientific, Waltham, MA, USA) conjugated to a chimeric anti-CEA antibody. FGS was performed using the MVX-10 fluorescence-dissecting microscope (Olympus) through a long-pass green fluorescent protein filter [36].

Several fluorescent antibody–dye conjugates have been evaluated in early-phase clinical trials for pancreatic cancer, reflecting growing interest in adopting FGS to the operating room. In a recent study, Lu et al. used an anti-epidermal growth factor (EGFR) antibody panitumumab in 11 patients with PDAC and demonstrated that 50 mg of panitumumab-IRDye800CW preoperatively enhanced intraoperative disease visualization with no adverse effects [24]. In 2021, Meijer et al. utilized SGM-101, an antibody specific to carcinoembryonic antigen (CEA) conjugated to a 700 nm fluorescent dye, to effectively detect pancreatic and liver metastasis during oncologic resection in 11 patients [37]. These clinical trials are excellent examples of the safety and potential improvement that FGS offers in oncologic surgery. A list of fluorescent probes used in clinical trials for PDAC is shown in Table 1.

In the present study, we used a clinically available fluorescence laparoscope (Stryker 1688, Stryker Corporation, Kalamazoo, MI, USA) with SPY imaging to examine the translational potential of CA19-9-IRDye800CW for intraoperative detection of pancreatic tumors. CA19-9-IR800 accurately detected orthotopic pancreatic tumors under laparoscopic SPY fluorescence. While our initial imaging results using the Pearl imaging system are encouraging, the current findings using a clinically validated SPY laparoscope represent a significant advancement toward the clinical applicability of CA19-9 targeted surgical therapy.

The Pearl Trilogy system is a small animal NIR imaging platform commonly used for preclinical research. It allows for high-resolution imaging in a controlled environment, making it ideal for the initial validation of fluorescent probes such as CA19-9-IRDye800CW. However, the Pearl system is incompatible with human surgical procedures and lacks real-time operative capability. In contrast, the SPY system is an FDA-approved laparoscopic platform currently used in clinical practice. By demonstrating the effectiveness of CA19-9-IRDye800CW using this platform, we provide direct evidence supporting its potential clinical adoption. The shift from a preclinical imaging system such as the Pearl to a clinically relevant surgical tool (SPY) is critical in translational research. These promising results support the application of CA19-9-IRDye800CW in FGS for laparoscopic detection and resection of pancreatic cancer—a field still in its early stages but poised to redefine oncologic surgery by enhancing intraoperative decision-making and improving overall patient outcomes and survival. However, translating fluorescent imaging agents from preclinical models to humans presents several challenges. These include potential immunogenicity of antibody-based probes, differences in pharmacokinetics and tissue penetration in larger organisms, and reduced signal-to-noise ratios in human tissues. Additionally, regulatory pathways for clinical approval of such agents remain complex and require extensive safety and efficacy data.

While our results are promising, several limitations should be noted. While sample sizes were appropriate for initial feasibility, particularly in treatment arms, the number of control animals was limited and may reduce statistical power. A larger sample size evaluated under SPY fluorescence would enhance statistical power and translational relevance. The cell lines used in the present study are widely used for experiments of this nature [38,39]. Inclusion of additional pancreatic cancer cell lines would further strengthen the generalizability of findings. Moreover, the absence of functional or long-term outcome assessments, such as margin status, recurrence, or survival, limits direct evaluation of clinical benefit. Given the heterogeneity of PDAC, reliance on a single marker such as CA19-9 may limit sensitivity. Future studies should explore dual-targeting strategies using markers like CEA in conjunction with CA19-9, using spectrally distinct fluorophores. Future studies should incorporate additional imaging parameters such as signal-to-noise ratio (SNR), contrast-to-noise ratio (CNR), and absolute fluorescence intensity thresholds to further evaluate clinical applicability. These metrics are commonly used in human imaging validation studies and would complement the TPR and TBR presented in the present study.

## 5. Conclusions

The present study used a near-infrared 800-nanometer fluorophore conjugated to an anti-CA19-9 antibody, an important biomarker for pancreatic cancer, to detect pancreatic cancer tumors in orthotopic mouse models. The CA19-9-IRDye800CW conjugate was examined using NIR and SPY fluorescence, yielding promising results. Such improvements over previous studies should provide clinical benefit to pancreatic cancer patients.

## Figures and Tables

**Table 1:** Fluorescent probes used in clinical trials for pancreatic ductal adenocarcinoma.

Fluorescent Probe	Target	Fluorophore	Clinical Trial(s)	Clinical Trials.gov ID
SGM-101	Carcinoembryonic antigen (CEA)	700 nm fluorophore	Detection of pancreatic/liver metastases	NCT02784028 [25], NCT02973672 [26]
Cetuximab–IRDye800CW	Epidermal growth factor receptor (EGFR)	IRDye800CW	Intraoperative imaging in pancreatic and head/neck cancer	NCT02736578 [27]
Pnitunimab-IRDye800CW	Epidermal growth factor receptor (EGFR)	IRDye800CW	Tumor-specific fluorescence-guided surgery in pancreatic cancer	NCT03384238 [24]
Bevacizumab-IRDye800CW	Vascular endothelial growth factor (VEGF)	IRDye800CW	Fluorescence imaging in pancreatic cancer	NCT02743975 [28]

## Data Availability

The raw data supporting the conclusions of this article will be made available by the authors on request.

## Abbreviations

The following abbreviations are used in this manuscript:

CA19-9	Carbohydrate antigen 19-9
FGS	Fluorescence-guided surgery
IP	Intraperitoneal
mFI	Mean fluorescence intensity
NIR	Near-infrared
PBS	Phosphate-buffered saline
PDAC	Pancreatic ductal adenocarcinoma
TBR	Tumor-to-background ratio
TLR	Tumor-to-liver ratio
TPR	Tumor-to-pancreas ratio
